# Association of ABO blood groups with Chikungunya virus

**DOI:** 10.1186/1743-422X-7-140

**Published:** 2010-06-25

**Authors:** Naresh CVM Kumar, Mahathi Nadimpalli, Vishnu R Vardhan, Sai DVR Gopal

**Affiliations:** 1 Department of Virology, College of Sciences, Sri Venkateswara University, Tirupati 517502, India; 2 C R RAO Advanced Institute of Mathematics, Statistics and Computer Science (AIMSCS) - DST, University of Hyderabad Campus, Prof.C.R.Rao Road, Gachibowli, Hyderabad - 500 046, India

## Abstract

Chikungunya virus (CHIKV) an emerging arboviral infection of public health concern belongs to the genus *Alphavirus*, family *Togaviridae*. Blood group antigens are generally known to act as receptors for various etiological agents. The studies defining the relationship between blood groups and CHIKV is limited and hence it is necessary to study these parameters in detail. In the present study 1500 subjects were enrolled and demographic data (Age, Gender, Blood group, CHIKV infection status, and CHIKV infection confirmation mode) was collected from them. The risk of acquiring CHIKV disease and its association with factors such as blood group, age and gender was analyzed statistically. The data of this study showed a possible association between blood group, age and gender of the study population with CHIKV infection. It is observed that CHIKV infections were higher in individuals with Rh positive blood group when compared to their Rh negative counterparts.CHIKV infections were found to be higher in Rh positive individuals of AB and A blood groups than that of Rh negative counterparts. Results also indicated that infections were higher in adults belonging to the age group > 30 years and also higher in males as compared to females enrolled in this study. These data present further evidence for the association of the blood groups, age and gender to susceptibility to CHIKV infection. Further studies are needed to confirm these findings. This is the second study showing the possible association of blood groups with chikungunya.

## Findings

Chikungunya virus (CHIKV), belonging to the genus *Alphavirus*, family *Togaviridae *is transmitted by *Aedes *mosquitoes (*Ae. aegyptii and Ae. albopictus*). *Ae. albopictus *played a pivotal role in transmitting the CHIKV in the recent outbreaks of Reunion islands, North Eastern Italy and India [1-3]. The disease is usually characterized by fever, arthralgia and myalgia. It was first reported from Makonde plateau, Tanzania during 1952-53 and since then, has been held responsible for explosive epidemics in Africa, India and South East Asia [4,5]. CHIKV, an enveloped virus containing positive sense single stranded RNA as its genetic material is about 11,805 nucleotides in length containing ORF1 and ORF2 of about 7422 and 3744 nucleotides in length respectively. Phylogenetic analysis based on partial E1 gene sequences showed the presence of three distinct CHIKV phylogroups. The first phylogroup contained all isolates from West Africa; the second phylogroup involved isolates from East, Central and South Africa (ECSA); and the third phylogroup contained Asian isolates. CHIKV one of the six major vector borne diseases endemic to India has reemerged causing severe mortality during the recent outbreak [6]. It has been declared as a high priority pathogen by NIH [7]. It affected 216 districts in India with an estimated 1.38 million people as its victims by the end of 2006 and which further declined to an estimated 59,535, 95,091 and 68,245 cases by the end of 2007, 2008 and 2009, respectively [8,9]. Andhra Pradesh, the most affected state was first to report the CHIKV epidemic in December 2005 in India [10]. RT-LAMP assay is a valuable tool used for rapid, real-time detection as well as quantification of CHIKV acute phase samples and will serve as an important tool for CHIKV surveillance in developing countries [11]. The association of diseases with blood groups has always been under controversial discussion. The relationship of blood groups of population and their susceptibility to diseases like plague, small pox, malaria, cholera etc suggest the possible role of blood group antigen in the occurrence of diseases. During the past twenty years there are many reports documenting the role of blood group antigens as receptors for parasites, bacteria and viruses [12]. Association of blood groups with HBV infection and fibrosis severity in HCV infection has been reported earlier [13]. The studies defining the relationship between blood groups and CHIKV is limited and hence it is necessary to study these parameters in detail. Keeping this in view the present study was designed to determine the relationship between CHIKV infection and ABO/Rhesus blood groups, age and gender.

During January-February 2010, 1500 subjects from Sri Venkateswara University Campus, Andhra Pradesh, India were enrolled in the study. A questionnaire was prepared and demographic data recorded for each patient included age, gender, blood group, CHIKV infection status (Yes/No) and CHIKV infection confirmation mode (Self declared CHIKV cases (SDC)/CHIKV infection confirmed by a physician). The importance of the present study was explained to the patients and oral consent from all the patients were obtained prior to data collection. The statistical tools such as, Odds ratio (OR) with 95% confidence interval (C.I) is presented to know the likelihood of acquiring CHIKV disease with respect to different blood groups: O, A, B and AB (Both Rh positive and Rh Negative individuals) when compared to healthy individuals. Chi-square test (χ^2 ^test) statistic was also carried out to observe the association between the blood groups and CHIKV infection. A p value < 0.05 was considered to be statistically significant.

**Figure 1:**
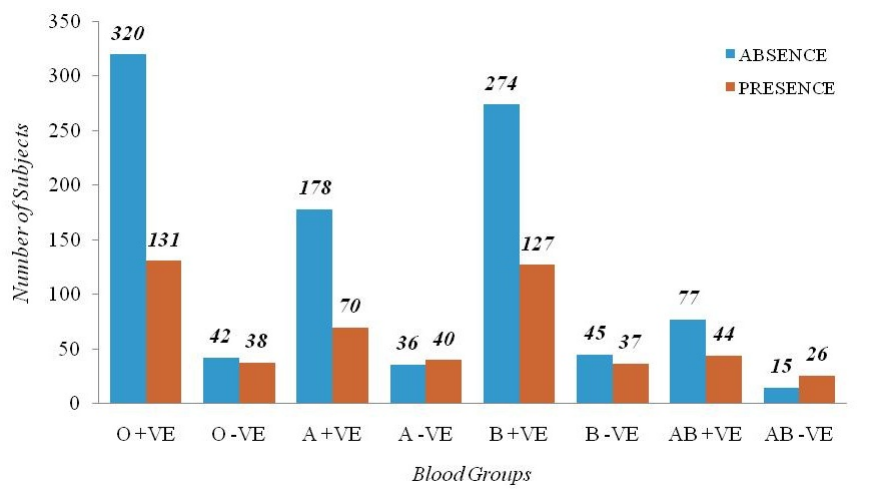
**Representation between various blood groups and CHIKV disease of the present study**.

**Figure 2:**
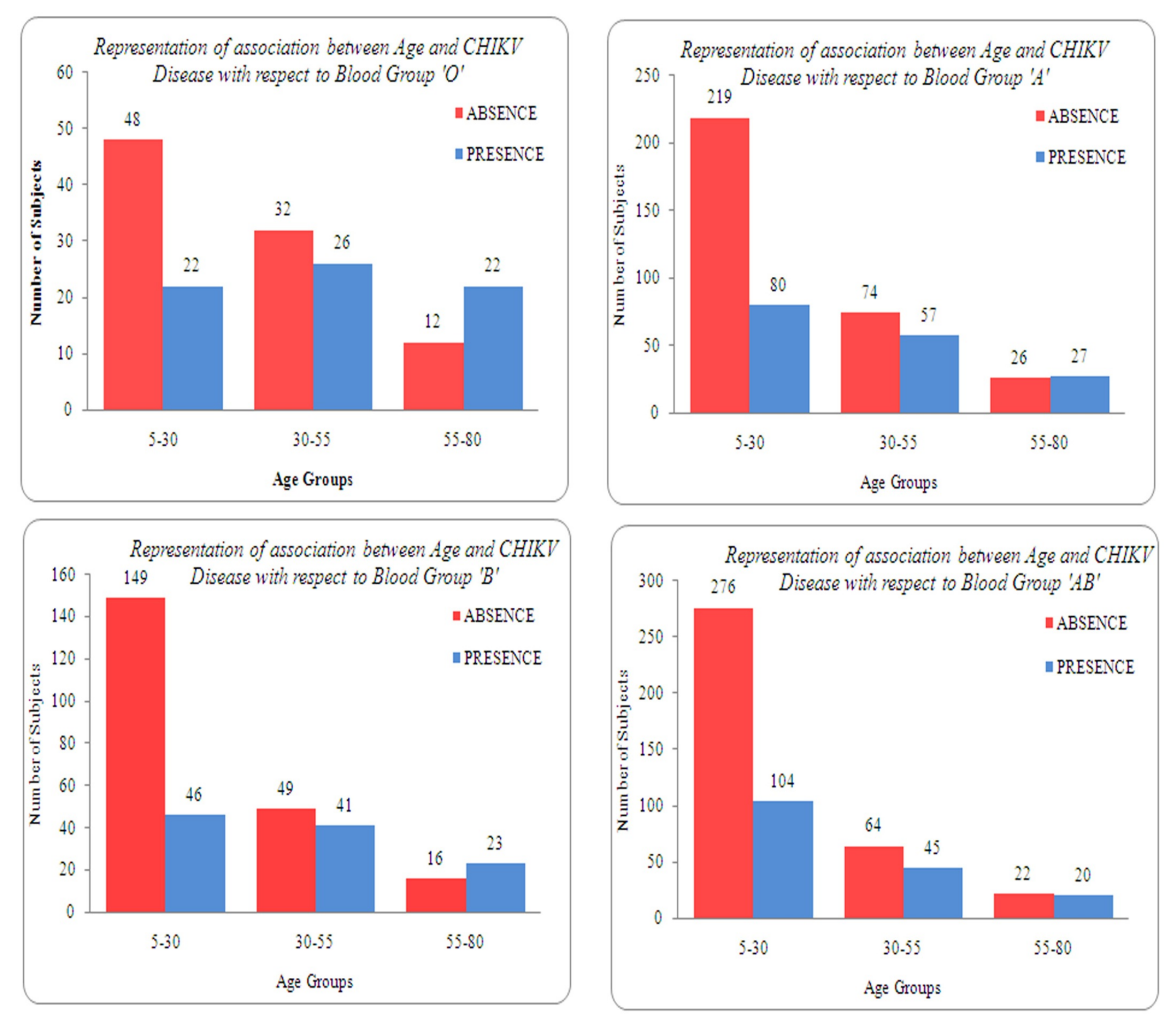
**Representation of association between Age group and CHIK disease**.

Among 1500 subjects enrolled in the study, 860 (57.3%) were males and 640 (42.7%) were females. Among them 513(34.2%) reported to have acquired CHIKV infection and 987(65.8%) individuals reported absence of CHIKV fever. Among these 513 individuals 409 (79.7%) patients were previously confirmed of CHIKV infection by Medical Officer, S.V.University Health Center by RT-PCR/IgM strip analysis carried out at Viral diagnostic Laboratory, Department of Virology, S.V.University, Tirupati as described by us earlier [14]. The remaining 104 (20.27%) individuals self declared to have acquired CHIKV infection (SDC) according to the CHIKV suspected case definition by NICD, New Delhi [15]. Based on blood group antigens the individuals were kept in four groups A (324), B (483), AB (162) and O (531). Figure 1 depicts the representation between various blood groups and CHIKV disease of the present study. The cross tabulation between the Rh positive and Rh Negative subjects of different blood groups were associated with the CHIKV status (Table 1). χ^2 ^test results showed the statistical significance at p < 0.05, which suggests that there exists an association between the blood groups and the risk of acquiring CHIKV. The Odds ratio is one of the familiar statistical measures used to know the likelihood of possessing the risk of acquiring CHIKV disease. The same measure is used here to know the likelihood of acquiring CHIKV disease between Rh positive and Rh negative individuals. The likelihood of acquiring CHIKV disease by O positive subjects was 2.2 times more than O negative individuals ie., OR_O _= 2.210 (95% C.I: 1.363, 3.585). The likelihood of acquiring CHIKV disease by A positive subjects is 2.8 times more than A negative subjects i.e. OR_A _= 2.825 (95% C.I: 1.666, 4.792). In the same manner the likelihood of acquiring CHIKV disease by B positive and AB positive subjects is 1.7 & 3 times more than B negative and AB negative subjects OR_B _= 1.774 (95% C.I: 1.094, 2.876) and OR_AB _= 3.033 (95% C.I: 1.454, 6.329)respectively. It is also observed that in A and AB blood groups, though the Rh positive and Rh negative subjects are almost proportionate in number, but the likelihood of acquiring CHIKV disease is more to Rh positive subjects. The association is observed statistically between blood groups and status of CHIKV focusing on the 'age' factor (Table 2). χ^2 ^test results showed statistically significant differences at p < 0.05. The status of CHIKV across the age distribution is presented for all the blood groups (Figure 2). The individuals > 30 age group are found to be more susceptible in acquiring CHIKV disease. It is also observed that the association between gender and the status of CHIKV is statistically significant (χ^2 ^= 5.926, p < 0.05) and the Odds Ratio_Male/Female _= 1.306 (95% C.I: 1.053, 1.619). Therefore male individuals are 1.3 times more susceptible in acquiring CHIKV disease when compared to their female counterparts.

**Table 1:** Cross tabulation between the Rh positive and Rh Negative subjects of different blood groups with the CHIKV status

Blood group	Status of Chikungunya		Total	Chi-square,	Odds Ratio
				
	ABSENCE	PRESENCE		p-value	(95% C.I)
O +VE	320	131	451	10.664,	2.210
		
O -VE	42	38	80	p = 0.001*	(1.363, 3.585)
		
**Total**	**362 (68.17%)**	**169 (31.83%)**	**531 (100%)**		

A +VE	178	70	248	15.452,	**2.825**
		
A -VE	36	40	76	p = 0.000*	**(1.666, 4.792)**
		
**Total**	**214 (66.05%)**	**110 (33.95%)**	**324 (100%)**		

B +VE	274	127	401	5.493,	1.774
		
B -VE	45	37	82	p = 0.019*	(1.094, 2.876)
		
**Total**	**319 (66.05%)**	**164 (33.95%)**	**483 (100%)**		

AB +VE	77	44	121	9.132,	**3.033**
		
AB -VE	15	26	41	p = 0.003*	**(1.454, 6.329)**
		
**Total**	**92 (56.79%)**	**70 (43.20%)**	**162 (100%)**		

'*' represents significance at 0.05 level; CI, Confidence Interval

**Table 2:** Association between age group and CHIKV status

Blood Group	Age group	Status of Chikungunya		Total
			
		ABSENCE	PRESENCE	
O	5-30	48	22	70
	
Chi-square = 12.802*	30-55	32	26	58
	
p = 0.002	55-80	12	22	34
	
	Total	92 (56.8%)	70 (43.2%)	162 (100.0%)

A	5-30	219	80	299
	
Chi-square = 25.631*	30-55	74	57	131
	
p = 0.000	55-80	26	27	53
	
	Total	319 (66.0%)	164 (34.0%)	483 (100%)

B	5-30	149	46	195
	
Chi-square = 19.066*	30-55	49	41	90
	
p = 0.000	55-80	16	23	39
	
	Total	214 (66.0%)	110 (34.0%)	324 (100%)

AB	5-30	276	104	380
	
Chi-square = 10.424*	30-55	64	45	109
	
p = 0.005	55-80	22	20	42
	
	Total	362 (68.2%)	169 (31.8%)	531 (100%)

'*' represents significance at 0.05 level

In conclusion our results implicate that blood group, age and gender are found to be associated with the status of CHIKV. It is observed that the Rh positive individuals are more susceptible when compared to their Rh negative counterparts in acquiring CHIKV disease. Using odds ratio (OR), the differences can be observed in between the Rh positive individuals and Rh negative individuals of AB and A blood groups followed by O and B blood groups, where the Rh positive individuals are likely to have more chance of acquiring CHIKV disease than that of Rh negative individuals. The interpretation on risk of acquiring CHIKV disease is given based on the OR value of each blood group with respect to their Rh positive and Rh negative blood groups. However, these results do not comply with the previously reported results where O positive individuals were shown to be more susceptible in acquiring CHIKV disease [16]. The difference in the association of the blood groups and CHIKV infection of the present study to that of the previously documented report emphasizes the necessity of carrying out similar studies at multicentre level in order to generate more data. With increase in age (>30), the chances of acquiring CHIKV disease are more. Movement of people outdoors during day time when the activity of *Ae.albopictus *is at its peak, lesser personal protection (towards mosquitoes) and individual's differential immune response to diseases are some of the speculative reasons for increased susceptibility to CHIKV infection in higher age groups (> 30 years)[1,17]. In the present study higher CHIKV infection rates were observed in males compared to their female counterparts, an observation which was also evidenced earlier in North eastern Italy and Southern India and differential exposure to *Ae.albopictus *mosquitoes is supposed to be the plausible reason [1,18]. To the best of our knowledge, our study is the second report analyzing the possibility of an association of blood groups with CHIKV seropositivity. Identification of human cell surface receptor(s) for CHIKV is quite necessary for further understanding its pathophysiology in humans [19].Molecular and functional studies will necessarily be helpful in disclosing the association of blood group antigens and CHIKV infections. The role of blood group antigens as important key factors in disease development is evident from earlier studies [12] and hence their possible role on CHIKV susceptibility cannot be ruled out and needs further investigation.

## Competing interests

The authors declare that they have no competing interests.

## Authors' contributions

CVMNK designed and performed the experiment. CVMNK also performed statistical analysis, interpreted the statistical data and drafted the manuscript. NM performed the experiment and contributed in writing the manuscript. RVV has done the statistical analysis and interpretation of the results. DVRSG participated in the design and coordination of the study, analysis of the data and drafting of the manuscript. All authors read and approved the final manuscript.
